# Use of intravenous lipid emulsion therapy as a novel treatment for brevetoxicosis in sea turtles

**DOI:** 10.1038/s41598-021-03550-y

**Published:** 2021-12-17

**Authors:** Justin R. Perrault, Heather W. Barron, Christopher R. Malinowski, Sarah L. Milton, Charles A. Manire

**Affiliations:** 1 Loggerhead Marinelife Center, Juno Beach, FL 33408 USA; 2 Clinic for the Rehabilitation of Wildlife, Sanibel, FL 33957 USA; 3 Purdue University, West Lafayette, IN 47907 USA; 4 Florida Atlantic University, Boca Raton, FL 33445 USA

**Keywords:** Physiology, Diseases

## Abstract

The southwest coast of Florida experiences annual red tides, a type of harmful algal bloom that results from high concentrations of *Karenia brevis*. These dinoflagellates release lipophilic neurotoxins, known as brevetoxins, that bind to sodium channels and inhibit their inactivation, resulting in a variety of symptoms that can lead to mass sea turtle strandings. Traditional therapies for brevetoxicosis include standard and supportive care (SSC) and/or dehydration therapy; however, these treatments are slow-acting and often ineffective. Because red tide events occur annually in Florida, our objective was to test intravenous lipid emulsion (ILE) as a rapid treatment for brevetoxicosis in sea turtles and examine potential impacts on toxin clearance rates, symptom reduction, rehabilitation time, and survival rates. Sea turtles exhibiting neurological symptoms related to brevetoxicosis were brought to rehabilitation from 2018–2019. Upon admission, blood samples were collected, followed by immediate administration of 25 mg ILE/kg body mass (Intralipid® 20%) at 1 mL/min using infusion pumps. Blood samples were collected at numerous intervals post-ILE delivery and analyzed for brevetoxins using enzyme-linked immunosorbent assays. In total, nine (four subadults, one adult female, four adult males) loggerheads (*Caretta caretta*), five (four juvenile, one adult female) Kemp’s ridleys (*Lepidochelys kempii*), and four juvenile green turtles (*Chelonia mydas*) were included in this study. We found that plasma brevetoxins declined faster compared to turtles that received only SSC. Additionally, survival rate of these patients was 94% (17/18), which is significantly higher than previous studies that used SSC and/or dehydration therapy (47%; 46/99). Nearly all symptoms were eliminated within 24–48 h, whereas using SSC, symptom elimination could take up to seven days or more. The dosage given here (25 mg/kg) was sufficient for turtles in this study, but the use of a higher dosage (50–100 mg/kg) for those animals experiencing severe symptoms may be considered. These types of fast-acting treatment plans are necessary for rehabilitation facilities that are already resource-limited. Intravenous lipid emulsion therapy has the potential to reduce rehabilitation time, save resources, and increase survival of sea turtles and other marine animals experiencing brevetoxicosis.

Harmful algal blooms occur worldwide and due to impacts to both humans and wildlife, monitoring efforts have increased in recent years^1^. In Florida USA, the most prominent harmful algal species is the dinoflagellate *Karenia brevis*, which produces potent neurotoxins known as brevetoxins^2–5^. Typically, *K. brevis* is present at low concentrations, but during favorable environmental conditions (e.g. currents, temperature, winds), these algae may multiply rapidly and turn the water reddish-brown^6,7^. Brevetoxins released from *K. brevis* are known to cause stranding and mortality in numerous wildlife species including fishes, sea turtles, sea birds, and marine mammals^8^ primarily through ingestion of contaminated food, leading to biomagnification up the food chain^5,9^. Other routes of exposure include drinking of contaminated water or inhalation of aerosolized toxins^4^.

Once ingested, brevetoxins act as potent neurotoxins that bind to sodium channels and inhibit their inactivation. This results in gastrointestintal disorders and loss of muscle coordination, partial to complete paralysis, circling behavior, and generalized lethargy, the latter of which impacts the ability to swim and breathe^10–12^. Additional effects include alterations in overall health and immune function and transfer of these toxins to offspring^13–18^. In particular, sea turtles exposed to red tide toxins often drown; however, if they are found to have stranded alive, they can sometimes be treated and saved at rehabilitation facilities.

Current treatment regimens of affected sea turtles include removal of the animal from the source of brevetoxins, dehydration therapy, and standard and supportive care (SSC)^12,19^. Therapeutic dehydration of affected turtles has shown some promise, but turtles are slow to recover and treatment has been successful in only a portion of the animals^12,19^. Previous studies have shown that total clearance time of the toxins in blood plasma with currently available treatments can take up to 80 days post-exposure^5^. In the last decade, physicians and veterinarians have begun to use intravenous lipid emulsion (ILE) in the treatment of various acute intoxications^20–22^. It is effective against lipid soluble agents (e.g. brevetoxins^10^), likely as a result of the expanded, intravascular “lipid sink”, which either draws toxins out of tissues allowing them to bind to lipids in the bloodstream or prevents toxins from reaching target tissues^21,23,24^. Other hypothesized mechanisms of action include energetic and metabolic effects^25,26^. Brevetoxins bound to the lipids from ILE would then likely be eliminated through the liver, bile, and feces^27,28^. A recent brevetoxin-exposure study using red eared sliders (*Trachemys scripta elegans*) as a model for sea turtles showed ILE to be highly effective at rapidly eliminating symptoms (i.e. within 6–24 h) and at removing brevetoxins from the bloodstream with no adverse effects due to the treatment^12^; however, the effectiveness of ILE in sea turtles naturally exposed to brevetoxins remains unknown.

The objectives of this study were to (1) document plasma brevetoxin concentrations using enzyme-linked immunosorbent assays (ELISA) before and after administration of ILE in three sea turtle species (loggerheads, *Caretta caretta*; Kemp’s ridleys, *Lepidochelys kempii*; green turtles, *Chelonia mydas*), (2) determine the effectiveness of ILE at reducing plasma brevetoxin concentrations and symptoms of brevetoxicosis, and (3) compare rehabilitation time and survival rates of sea turtles given ILE to those provided with SSC.

## Results

### Sample animals

Sea turtles included in this study stranded on Florida’s west coast from 27 Mar–21 Aug 2018 (*N* = 14) and from 9 Oct–27 Nov 2019 (*N* = 4). Nine loggerheads (four subadults, one adult female, four adult males) ranging from 47.8–132.4 kg and 75.0–99.3 cm standard straight carapace length (SCL), five Kemp’s ridleys (four juveniles, one adult female) ranging from 7.9–32.3 kg and 38.0–63.7 cm SCL, and four juvenile green turtles ranging from 6.2–32.9 kg and 36.7–63.2 cm SCL were sampled (see Supplemental Table S1 for description of stranding date, release date, life-stage class, sex, mass and SCL on intake, body condition score, clinical symptoms of brevetoxicosis upon admission, and additional treatments). Animals were treated as necessary for other secondary diseases/conditions (treatment/drug include parenthetically) including infection (ceftazidime), dehydration (crystalloid fluid based on plasma chemistry analysis), hypoglycemia (dextrose), gastrointestinal ailments (crystalloid fluid), anemia/malnutrition (iron/B12), spirorchiid infection (praziquantel), and metabolic acidosis (sodium bicarbonate).

### ELISA brevetoxin concentrations

**Figure 1:**
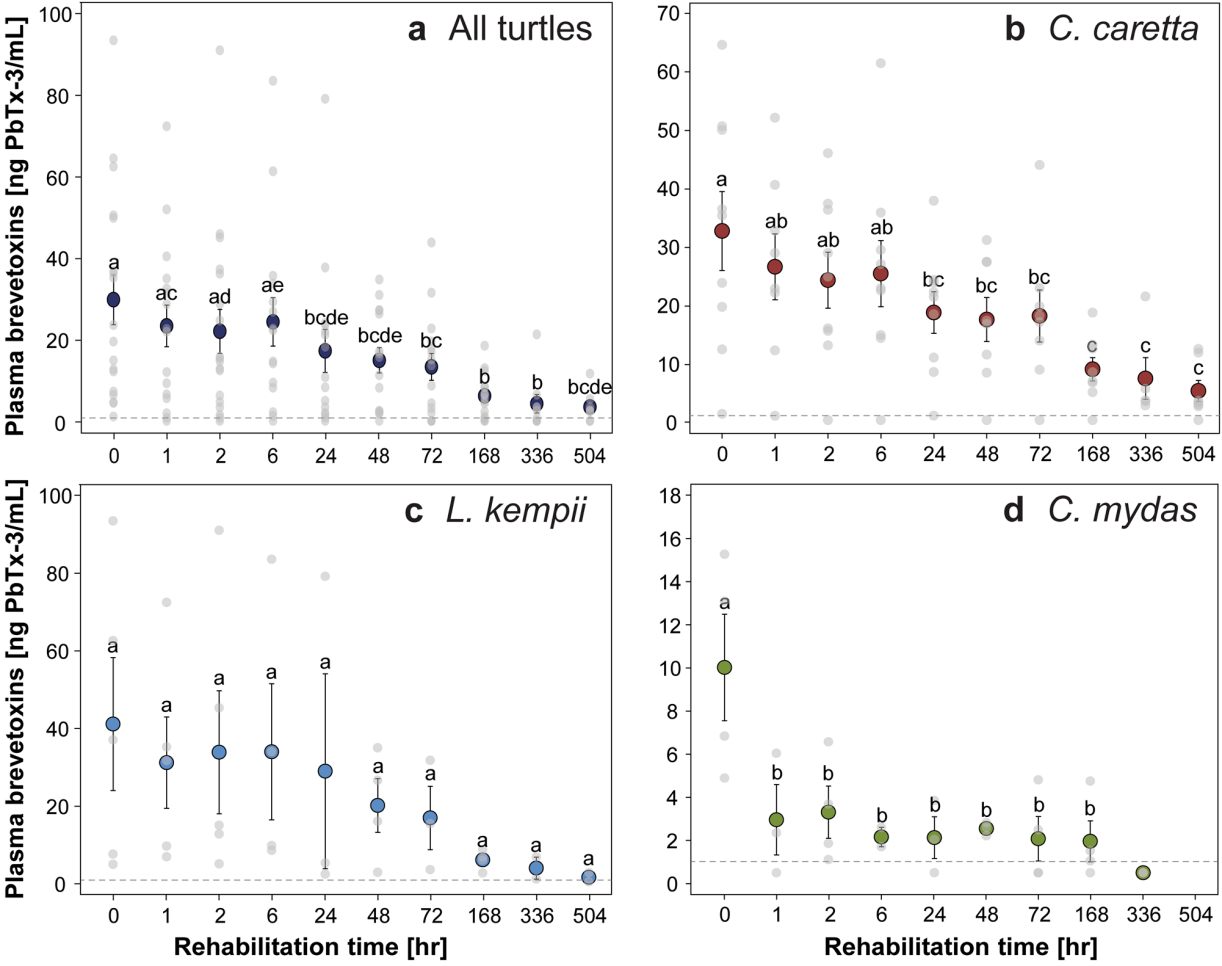
Clearance of plasma brevetoxins in (**a**) all turtles combined, (**b**) loggerhead (*Caretta caretta*), (**c**) Kemp’s ridley (*Lepidochelys kempii*), and (**d**) green (*Chelonia mydas*) sea turtles undergoing rehabilitation for brevetoxicosis. All turtles included in the figure received intravenous lipid emulsion after an initial sample was taken at admission (T = 0 h). Each colored circle represents the mean ± standard error for all turtles at that time point. Light gray circles indicate the raw data. Note that the x-axis is not set to scale, but instead serves to show changes in concentrations through time. The horizontal gray-dashed lines represent the detection limit of the ELISA at 1 ng PbTx-3/mL. Different letters above each point represent significant differences.

Upon admission, brevetoxin concentrations in loggerheads, Kemp’s ridleys, and green turtles ranged from 1.6–64.6 ng PbTx-3/mL, 5.0–93.4 ng PbTx-3/mL, and 4.9–15.3 ng PbTx-3/mL, respectively (Table 1). A Kruskal–Wallis test revealed no species-specific differences in concentrations upon admission (*P* > 0.05). After administration of ILE (i.e. at time 1 h), mean brevetoxin concentrations dropped by 6.1 ng PbTx-3/mL (18.6%) in loggerheads, 10.0 ng PbTx-3/mL (24.2%) in Kemp’s ridleys, and 7.1 ng PbTx-3/mL (70.5%) in green turtles, on average (Fig. 1, Table 1). The largest decrease in concentrations for loggerheads and Kemp’s ridleys occurred at the 168 h sample, with concentrations falling by 23.6 ng PbTx-3/mL (90.1%) and 38.6 ng PbTx-3/mL (93.8%), on average, respectively, in comparison to brevetoxin concentrations on intake. The largest decrease in concentrations for green turtles occurred at the one-hour post ILE delivery time point (Fig. 1, Table 1), whereby concentrations fell by 7.0 ng PbTx-3/mL (70.1%). Brevetoxin concentrations at each subsequent time point and percent change are also reported in Table 1. Plasma brevetoxin concentrations in sea turtles in the literature are also provided for comparison to results of this study (Table 2).

**Table 1:** Brevetoxin concentrations (ng PbTx-3/mL) at each sampling point in rehabilitating loggerhead (*Caretta caretta*), Kemp’s ridley (*Lepidochelys kempii*), and green (*Chelonia mydas*) sea turtles that stranded during a red tide bloom event.

Species	Time point (h)	Mean ± SD	Median	Range	*N*	# BDL	TD (%)	TPD (%)
*C. caretta*	0	32.8 ± 20.3^a^	35.5	1.6–64.6	9	0	–	–
	1	26.7 ± 15.9^ab^	26.0	1.2–52.2	8	0	− 18.6%	− 18.6%
	2	24.4 ± 14.4^ab^	25.0	< 1.0–46.1	9	1	− 25.6%	− 8.6%
	6	25.5 ± 16.9^ab^	23.0	< 1.0–61.5	9	1	− 22.2%	4.6%
	24	18.9 ± 10.7^bc^	21.5	1.3–38.0	9	0	− 42.4%	− 26.0%
	48	17.7 ± 10.6^bc^	17.2	< 1.0–31.3	8	1	− 46.1%	− 6.4%
	72	18.3 ± 12.6^bc^	17.8	< 1.0–44.1	8	1	− 44.2%	3.5%
	168	9.2 ± 5.7^c^	7.9	< 1.0–18.9	8	1	− 72.0%	− 49.8%
	336	7.6 ± 7.9^c^	3.9	3.0–21.6	5	0	− 76.8%	− 17.1%
	504	5.5 ± 4.8^c^	3.5	< 1.0–12.6	7	1	− 83.3%	− 27.9%
	505–599	–	3.3	–	1	0	− 90.1%	− 40.8%
	600–699	–	2.0	–	1	0	− 93.9%	− 38.2%
	800–899	1.8 ± 0.4	1.8	1.5–2.1	2	0	− 94.4%	− 9.0%
	1000–1099	–	1.4	–	1	0	− 95.8%	− 24.6%
	1100–1199	–	1.5	–	1	0	− 95.5%	8.0%
	1200–1299	–	1.2	–	1	0	− 96.4%	− 20.1%
	1400–1499	–	1.5	–	1	0	− 95.5%	25.2%
*L. kempii*	0	41.2 ± 37.5^a^	37.1	5.0–93.4	5	0	–	–
	1	31.2 ± 26.3^a^	31.7	7.0–72.5	5	0	− 24.2%	− 24.2%
	2	33.9 ± 35.4^a^	15.1	5.1–45.3	5	0	− 17.7%	8.5%
	6	34.0 ± 35.1^a^	21.9	8.6–83.6	4	0	− 17.4%	0.4%
	24	29.0 ± 43.5^a^	5.4	2.5–79.2	3	0	− 29.5%	− 14.7%
	48	20.2 ± 13.9^a^	21.4	3.0–35.0	4	0	− 51.0%	− 30.4%
	72	14.1 ± 14.1^a^	15.5	3.6–31.8	3	0	− 65.7%	− 30.0%
	168	2.5 ± 2.5^a^	6.5	2.8–8.9	4	0	− 93.8%	− 82.0%
	336	4.1 ± 4.0^a^	4.1	1.2–6.9	2	0	− 90.2%	59.5%
	504	1.7 ± 1.7^a^	1.7	< 1.0–2.8	2	1	− 95.9%	− 58.8%
*C. mydas*	0	10.0 ± 5.0^a^	10.0	4.9–15.3	4	0	–	–
	1	3.0 ± 2.8^b^	2.4	< 1.0–6.0	3	1	− 70.5%	− 70.5%
	2	3.5 ± 2.4^b^	2.9	1.3–6.7	4	0	− 65.6%	16.6%
	6	2.2 ± 0.6^b^	2.2	1.7–2.6	2	0	− 78.4%	− 37.4%
	24	2.1 ± 1.7^b^	2.1	< 1.0–3.9	3	1	− 78.7%	− 1.4%
	48	2.6 ± 0.3^b^	2.6	2.2–2.8	3	0	− 74.6%	19.7%
	72	2.1 ± 2.1^b^	1.5	< 1.0–4.8	4	2	− 79.2%	− 18.4%
	168	2.0 ± 1.9^b^	1.3	< 1.0–4.8	4	1	− 80.4%	− 5.8%
	336	–	< 1.0	–	3	3	− 95.0%	− 74.5%

All turtles, except one, included here were administered 25 mg/kg of intravenous lipid emulsion (one Kemp’s ridley was given a dose of 100 mg/kg) after time point = 0 h. Samples that fell below the limits of detection (1.0 ng PbTx-3/mL) were assigned to a value of half of the detection limit. Different superscript letters next to the means indicate significant differences. BDL, below limits of detection; TD, total difference; TPD, time-point difference.

**Table 2:** Plasma brevetoxin concentrations (ng PbTx-3/g or mL) in loggerhead (*Caretta caretta*), Kemp’s ridley (*Lepidochelys kempii*), and green (*Chelonia mydas*) sea turtles from the literature.

Species	Year	Status	Method	*N*	# BDL	Mean ± SD	Median	Range	Study
*C. caretta*	2006	Live stranded	ELISA	9	0	68.0 ± 30.7	77.0	34.0–89.0	^13^
	2005–2006	Live stranded	ELISA	34	5	32	21	< 1–107	^5^
	2014	Nesting	ELISA	48	0	9.1 ± 6.1	8.2	2.1–26.7	^17^
	2018–2019	Live stranded	ELISA	9	0	32.8 ± 20.3	35.5	1.6–64.6	This study
*L. kempii*	2005–2006	Live stranded	ELISA	5	1	63	78	< 1–82	^5^
	2012–2013	Wild caught	ELISA	9	0	22.6 ± 6.5	22.2	13.0–33.8	^16^
	2014	Wild caught	ELISA	21	6	2.6 ± 2.4	1.5	< 1.0–8.6	^18^
	2018–2019	Live stranded	ELISA	5	0	41.2 ± 37.5	37.1	5.0–93.4	This study
*C. mydas*	2005–2006	Live stranded	ELISA	6	3	1.2	< 1	1–4	^5^
	2014	Wild caught	ELISA	8	2	1.7 ± 1.5	1.3	< 1.0–5.2	^18^
	2015	Wild caught	UPLC-MS/MS^a^	21	21	< 75	–	–	^29^
	2018–2019	Live stranded	ELISA	4	0	10.0 ± 5.0	10.0	4.9–15.3	This study

Concentrations reported in plasma samples from the present study are those prior to administration of any additional treatments, including intravenous lipid emulsion (i.e. time point = 0 h).

BDL, below detection limit; ELISA, enzyme-linked immunosorbent assay; SD, standard deviation; UPLC-MS/MS, ultra-performance liquid chromatograph/tandem mass spectrometry.

^a^Samples were analyzed for specific congeners brevetoxin-B and brevetoxin-3.

The linear mixed-effect model with all species combined revealed that species (*F*_2,13.1_ = 4.86, *P* = 0.026) and time point (*F*_1,123.3_ = 5.27, *P* = 0.023) had a statistically significant effect on brevetoxin concentrations, but not sex/maturity status (*F*_2,12.8_ = 0.91, *P* = 0.428; Fig. 1a, Supplemental Table S2). For linear mixed-effect model analysis in loggerheads, time point (*F*_1,16.3_ = 4.73, *P* = 0.045), but not sex/maturity (*F*_2,20.4_ = 0.18, *P* = 0.837) had a significant effect (Fig. 1b, Supplemental Table S3). For Kemp’s ridleys, the main effect sex/maturity was removed from the model because nearly all samples were from immature individuals. The main effect time point was significant in the model (*F*_1,10.6_ = 8.91, *P* = 0.013); however, with Tukey adjustments for multiple comparisons between time points, no significant differences occurred (Fig. 1c, Supplemental Table S4). For green turtles, the main effect sex/maturity was removed from the model because all samples were from immature individuals. The main effect time point was significant in the model (*F*_1,27.0_ = 5.25, *P* = 0.030; Fig. 1d, Supplemental Table S5).

**Figure 2:**
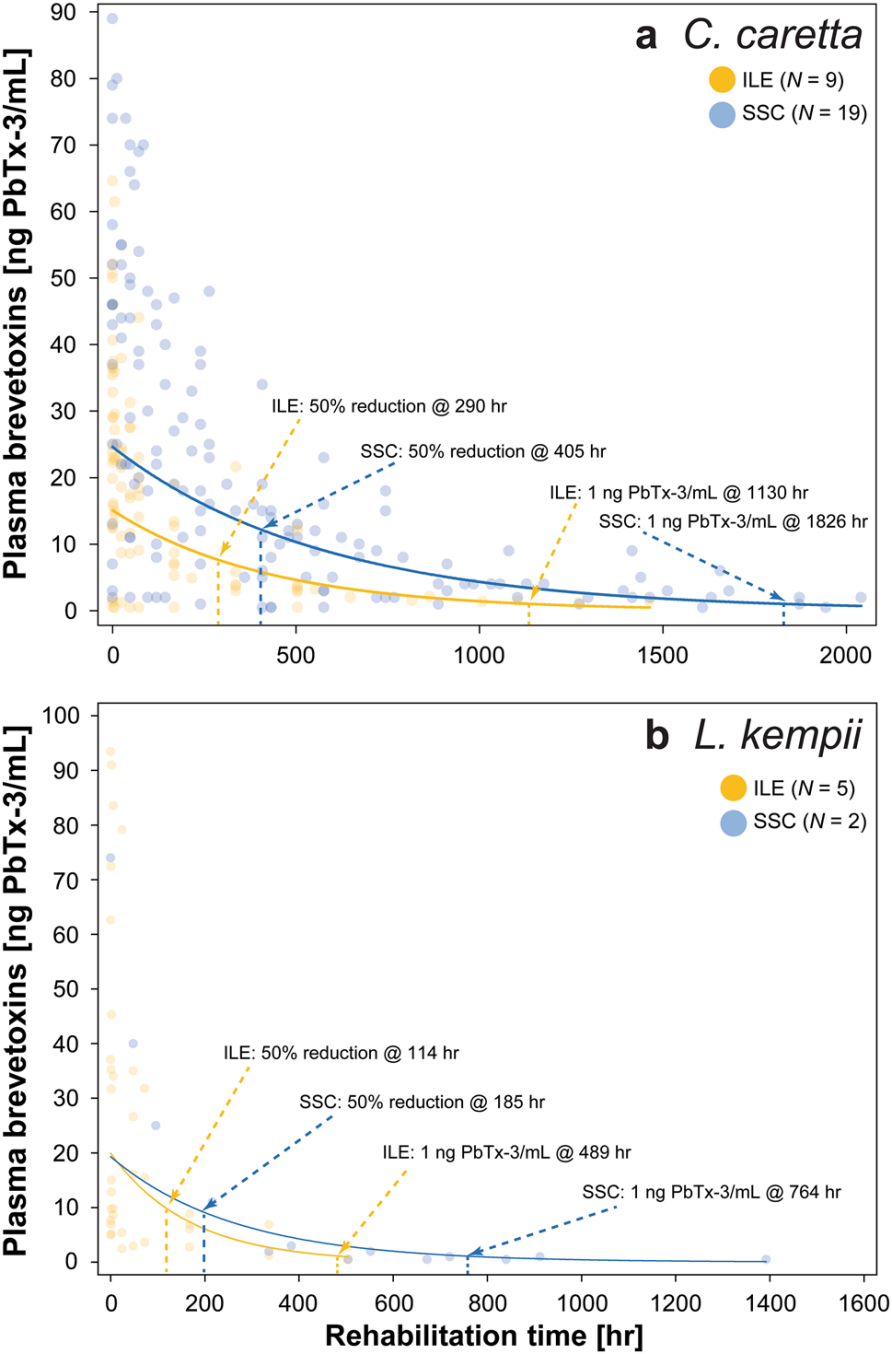
Time-series of plasma brevetoxin concentrations determined using enzyme-linked immunosorbent assays (ELISA) in (**a**) loggerhead (*Caretta caretta*) and (**b**) Kemp’s ridley (*Lepidochelys kempii*) sea turtles receiving intravenous lipid emulsion (ILE: yellow points/line) versus those treated with standard and supportive care (SSC: blue points/line)^5^. Points represent raw data for each treatment method with the lines-of-best fit also shown. The locations on the line-of-best fit are indicated where (1) an approximate 50% reduction in toxin concentration occurred in relation to concentration at time 0 h (i.e. admission) and (2) an approximate concentration of 1 ng PbTx-3/mL (the detection limit of the ELISA) was reached, indicating near complete clearance of the toxins from the blood plasma.

Time-series plasma brevetoxin concentrations analyzed using ELISA in loggerheads and Kemp’s ridleys given ILE and those provided with SSC from a separate study^5^ were compared. Using the line-of-best-fit to determine potential differences in brevetoxin clearance rates between the two treatment methods, we estimated that loggerheads given ILE (*N* = 9) reduced ~ 50% of their plasma brevetoxin loads by ~ 290 h (~ 12 d), whereas loggerheads given SSC (*N* = 19) reduced ~ 50% of their plasma brevetoxin loads by ~ 405 h (~ 17 d). Using this same method, we estimated that loggerheads given ILE or SSC reached ~ 1 ng Pbtx-3/mL (the detection limit of the ELISA) at 1130 h (~ 47 d) or 1826 h (~ 76 d), respectively (Fig. 2a). For Kemp’s ridleys, we estimated that those given ILE (*N* = 5) reduced ~ 50% of their plasma brevetoxin loads by ~ 114 h (~ 5 d), whereas Kemp’s ridleys given SSC (*N* = 2) reduced ~ 50% of their plasma brevetoxin loads by ~ 185 h (~ 8 d). Using this same method, we estimated that Kemp’s ridleys given ILE or SSC reached ~ 1 ng Pbtx-3/mL at 489 h (~ 20 d) or 764 h (~ 38 d), respectively (Fig. 2b). Green turtles were not evaluated as sample sizes were too low (*N* = 4 for ILE; *N* = 1 for SSC).

**Figure 3:**
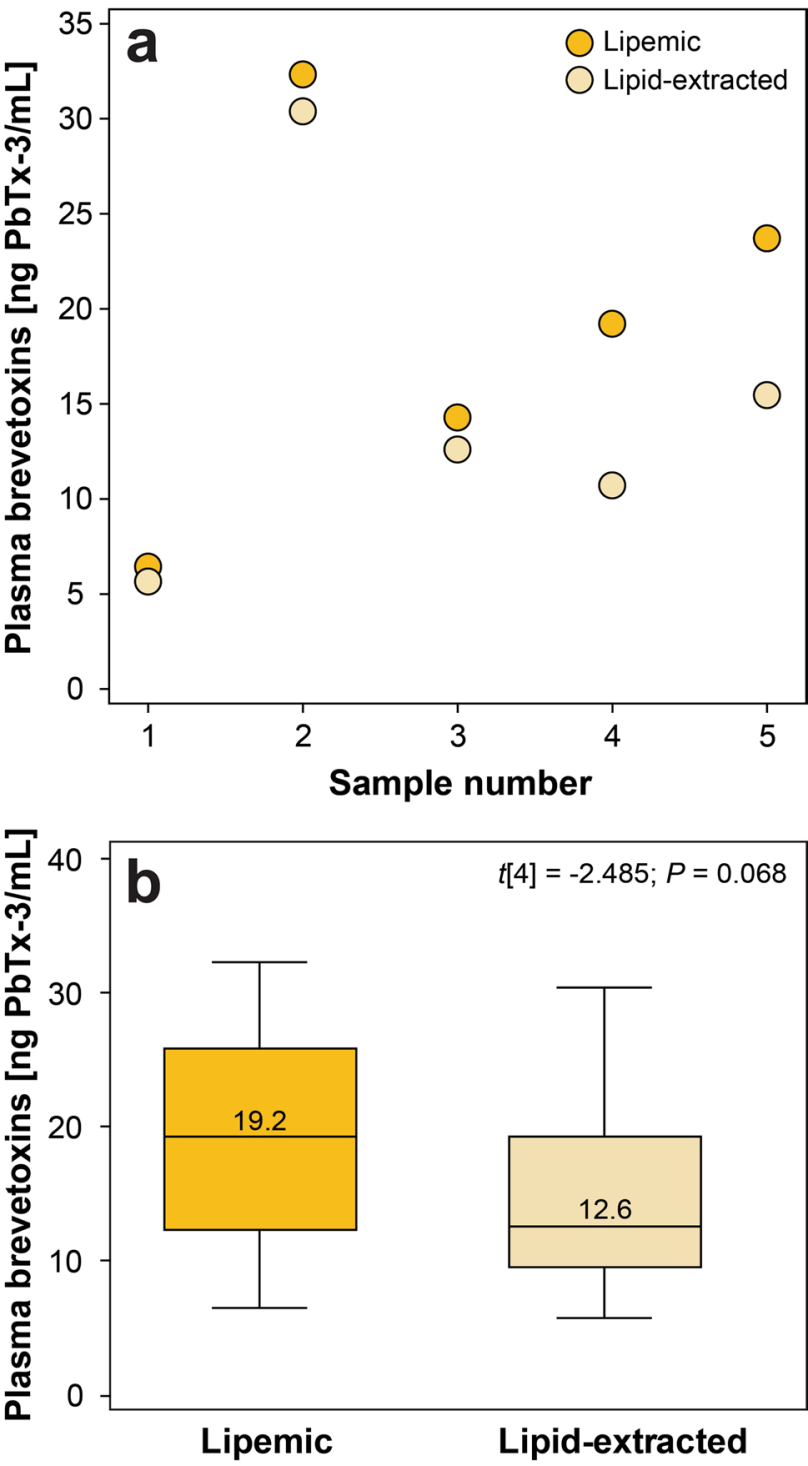
Plasma brevetoxin concentrations analyzed using ELISA in *N* = 5 lipemic samples compared to concentrations in the same samples after lipid-extraction by ultracentrifugation. Raw data are shown in (**a**), while box plots are shown in (**b**). The central yellow box represents the values from the lower to upper quartile (25th to 75th percentile), with the middle line representing the median (concentrations are shown). The vertical lines extend from the minimum to the maximum values. No outliers were present.

A paired-samples t-test revealed no significant difference (*t*[4] = -2.485; *P* = 0.068) between lipemic plasma samples (mean ± SD: 19.2 ± 9.7 ng PbTx-3/mL; range: 6.4–32.3 ng PbTx-3/mL) that were analyzed native (i.e. as is) versus those that were subjected to lipid extraction by ultracentrifugation (mean ± SD: 15.0 ± 9.3 ng PbTx-3/mL; range: 5.7–30.4 ng PbTx-3/mL) (Fig. 3).

### Symptom reduction, survival rate, and time in rehabilitation

Nearly all symptoms of brevetoxicosis (e.g. blink reflex, ability to hold up head and flippers, circling, clasper reflex, vent tone; Supplemental Table S1) improved or resolved within 24–48 h after administration of ILE, aside from conjunctival swelling, which was present for up to four days (96 h) post ILE. Using SSC, symptom elimination can take up to 7 days, or more^30,31^.

Survival rate of all patients included in this study was 94% (17/18), with species-specific survival rates reported in Table 3. Previous studies have documented survival rates of 72% (13/18; treated using SSC at CROW, 2016–2018)^32^, 40% (21/52; treated using SSC at Mote Marine Laboratory’s Sea Turtle Rehabilitation Hospital [MML STRH], Sarasota, FL, USA, 2005–2006)^5^, 32% (7/22; treated using SSC at MML STRH, 2005–2006), and 71% (5/7; treated using the diuretic furosemide: 5 mg/kg i.m. q 24 h at MML STRH, 2006)^19^. Fisher’s exact tests revealed that the percentage of turtles that survived rehabilitation using ILE therapy was not significantly different than those that received SSC at CROW (*P* = 0.177), but was significantly higher than turtles receiving SSC at MML STRH (*P* < 0.001 in both cases)^5,19^. No significant difference in survival was observed between turtles receiving ILE and those receiving furosemide (*P* = 0.180) (Table 3). Overall, combining all rehabilitation studies regarding brevetoxicosis in sea turtles, ILE showed a significantly higher survival rate (94%) than turtles receiving a combination of SSC and/or dehydration therapy (47%; *P* < 0.001)^5,19,32^.

**Table 3:** Survival rates and rehabilitation times of loggerhead (*Caretta caretta*), Kemp’s ridley (*Lepidochelys kempii*), and green (*Chelonia mydas*) sea turtles stranding due to brevetoxicosis from this study and the literature.

Species	# Survived	# Died	% Survival	Treatment	Days in rehab	Study
*C. caretta*	14	25	36% (14/39)	SSC	41–330^a^	^5^
	2	15	12% (2/17)	SSC	49–67	^19^
	5	2	71% (5/7)	D	41–87	^19^
	2	2	50% (2/4)	SSC	14–26	^32^
SSC & D	23	44	34% (23/67)	SSC & D	14–330	–
ILE	9	0	100% (9/9)	ILE	14–62	This study
*L. kempii*	5	1	83% (5/6)	SSC	81	^5^
	4	0	100% (4/4)	SSC	81–592^a^	^19^
	7	1	88% (7/8)	SSC	16–25	^32^
SSC	16	2	89% (16/18)	SSC	16–592	–
ILE	4	1	80%(4/5)	ILE	14–26	This study
*C. mydas*	2	5	29% (2/7)	SSC	34–325^a^	^5^
	1	0	100% (1/1)	SSC	198	^19^
	4	2	67% (4/6)	SSC	13–33	^32^
SSC	7	7	50% (7/14)	SSC	13–325	–
ILE	4	0	100% (4/4)	ILE	14–19	This study
ALL SSC & D	46	53	47% (46/99)	SSC & D	13–330	–
ALL ILE	17	1	94% (17/18)	ILE	14–62	–

Live stranded animals were brought to rehabilitation and provided with treatments including standard and supportive care (SSC; i.e. removed from source of toxins, placed in toxin-free water, provided with subcutaneous fluids, and gavage feeding), dehydration therapy (D; furosemide, 5 mg/kg i.m. q 24 h), or intravenous lipid emulsion (ILE; Intralipid 20%, 25–100 mg/kg). Survival rates from previous studies for each species were summed to provide comparisons to survival rates using ILE.

^a^Note that the release of some turtles was delayed due to the presence of secondary conditions or active red tide blooms in the area of rehabilitation.

Three turtles were transferred to other facilities for continued treatment and were not included in calculation of release times. Mean ± SD of release times for patients that received ILE was 23 ± 12 d (range: 14–62 d; *N* = 14), with species-specific release times reported in Table 3. Release times of turtles receiving ILE did not significantly differ (*P* = 0.504) from turtles provided with SSC at CROW (mean: 20 ± 6 d; range: 13–33 days; *N* = 11)^32^. Release times from other studies are also reported in Table 3; however, these results were not compared statistically due to low sample sizes and confounding factors associated with release (e.g. presence of secondary conditions or active red tide blooms in the area of rehabilitation, preventing immediate release)^5,19^.

#### Discussion

Red tides have been documented on Florida’s west coast since 1844 and similar to other harmful algal blooms, they appear to be increasing in frequency, duration, and geographic range; however it is unclear if this is due to nutrient runoff, aquaculture production, alterations in ocean temperatures, weather patterns, currents, and/or increased monitoring efforts^1,3,33–36^. On Florida’s west coast, red tide conditions (> 100,000 cells/L) have occurred in offshore waters in at least one county every year since 2000. On average, these blooms are present for 7 ± 3 months/year, with blooms lasting anywhere from 1–17 months^37^, often following periods of heavy rainfall from hurricanes and runoff associated with those storms^38^.

Large numbers of sea turtle strandings are attributed to red tides, with the majority consisting of loggerheads and Kemp’s ridleys^8,12,39^. Upon admission to rehabilitation, 100% (18/18) of sea turtles included in this study tested positive for brevetoxin exposure in plasma. In turtles from the present study, brevetoxin concentrations upon admission to rehabilitation did not significantly differ between species, likely due to low sample sizes; however, our results were similar to other studies^5,18^ whereby Kemp’s ridleys showed the highest mean brevetoxin concentrations (41.2 ng PbTx-3/mL), followed by loggerheads (32.8 ng PbTx-3/mL), and then green turtles (10.0 ng PbTx-3/mL). Differences in species-specific concentrations were expected and are likely related to dissimilarities in preferred prey items^5,8^ – loggerheads and Kemp’s ridleys forage on benthic mollusks and crustaceans, while green turtles feed primarily on seagrasses^40^. In sea turtles stranding in Texas during a red tide bloom, average brevetoxin concentrations in stomach and intestinal contents were lower in green turtles in comparison to Kemp’s ridleys^41^; however, the converse was true for sea turtles stranding dead in Florida, whereby green turtles had the highest mean concentrations in stomach contents and feces, followed by Kemp’s ridleys and then loggerheads^5^. Therefore, accumulation of brevetoxins likely differs by location and forage preference, resulting in differences in exposure^4,42,43^. Across studies, plasma brevetoxin concentrations in stranded sea turtles overlapped in range (Table 2), with highest mean concentrations in (1) loggerheads sampled during the bloom of Aug–Oct 2006^13^, (2) Kemp’s ridleys sampled during the blooms of 2005–2006^5^, and (3) green turtles from the present study. Any additional differences in exposure, despite large-scale blooms occurring across all studies, are likely related to localized *K. brevis* concentrations, location of stranding, avoidance behaviors, body mass and condition, habitat preference, and/or other physiological mechanisms^5,8,16,17^.

The use of ILE in turtles was first tested in an animal model (red-eared sliders, *T. scripta*) due to limitations with working with threatened and endangered sea turtles. In red-eared sliders experimentally dosed by intratracheal instillation with brevetoxin-3, animals showed typical symptoms of brevetoxicosis including muscle twitching, circling, head bobbing, paralysis, ataxia, and general lethargy. Within one hour post-ILE delivery, symptom reduction in the sliders occurred, with complete elimination of symptoms occurring within 6–24 h. Turtles given higher doses (100 mg/kg v. 50 mg/kg) experienced a faster reduction in symptoms, with no adverse effects noted in turtles receiving only ILE and no brevetoxin-3^28^. Due to the promising results with red-eared sliders, permitting to allow for ILE therapy trials in sea turtles was granted.

Due to the small sample size and large variation in plasma brevetoxin concentrations in Kemp’s ridleys across the sampling period in the present study, no statistical differences were observed (Fig. 1c); however, plasma brevetoxin concentrations significantly declined from concentrations upon admission in loggerheads at the 24 h sample (Fig. 1a) and in green turtles at the 1 h sample (Fig. 1d). Similar results were observed in dogs that ingested bromethalin, a neurotoxic rodenticide, whereby serum bromethalin concentrations declined from 4 to 1 ppb (75%) one hour after ILE delivery^44^. Additionally, in humans given bupivacaine followed by ILE, plasma concentrations were 20.6% and 14.3% lower 20 and 30 min after drug delivery, respectively, in comparison to a control solution^45^. However, ILE has been shown to have the opposite effect with other drugs, whereby toxicant concentrations were maintained or increased after administration^46^. We did not observe such rapid elimination of toxins after ILE delivery, which may be due to lower metabolic rates of sea turtles in comparison to endotherms^47^, as rats, mice, and seabirds exposed to brevetoxins had faster clearance times in comparison to sea turtles^48–50^. Brevetoxin concentrations did decline at a faster rate using ILE compared to SSC in loggerheads and Kemp’s ridleys (Fig. 2), whereby a 50% reduction in plasma concentrations was estimated to occur ~ 115 h (~ 5 d) and 71 h (~ 3 d) faster in loggerheads and Kemp’s ridleys receiving ILE, respectively. Additionally, clearance of plasma brevetoxins was estimated to occur ~ 696 h (~ 29 d) and 275 h (~ 12 d) faster compared to loggerheads and Kemp’s ridleys receiving only SSC, respectively^5^. In red-eared sliders receiving ILE, brevetoxins were also excreted at faster rates in bile and feces 24 h post-exposure in comparison to control animals^28^. It is possible that other treatments (e.g. fluid therapy) that turtles received for secondary conditions (e.g. dehydration) that were given concurrently with ILE impacted plasma brevetoxin concentrations, as intravenous fluids would likely dilute brevetoxin concentrations in plasma. Because gut passage time in sea turtles is relatively extensive (14 d or more)^51^, an additional method has been proposed to speed clearance of toxins using gut motility stimulating drugs to promote emptying of the intestine to eliminate toxins in the GI tract^19^.

Interestingly, brevetoxin concentrations increased at the one-hour time point, post ILE-delivery in one loggerhead and two Kemp’s ridleys. Additional increases were observed in at least one time point compared to previous time points in 17/18 turtles, with some time points showing higher concentrations on average in comparison to the previous time point (e.g. 2 h v 6 h time-points in loggerheads; Fig. 1; Table 1). Similar results were seen in one rehabilitating manatee stranding on Florida’s west coast due to brevetoxicosis, whereby brevetoxin concentrations increased in plasma three days after the initial sample was taken, and then subsequently declined 7 and 14 days after this initial increase^14^. Reasons for the observed increase in manatees were not discussed; however; radiographs from sea turtle patients included in this study showed the presence of food still in the digestive tract. Therefore, it is possible that concentrations increased in individuals at certain time points due to continued digestion of prey and reabsorption of brevetoxins from the gut^5,28^. An alternative explanation for this finding could be that after administration of ILE, brevetoxins are drawn out of cells and into extracellular compartments and subsequently into blood plasma, leading to increases in plasma concentrations post-ILE delivery.

A non-significant reduction in plasma brevetoxin concentrations was observed after lipid extraction using ultracentrifugation; however, results likely would have reached significance with a larger sample size, as all five samples subjected to ultracentrifugation showed lower concentrations in comparison to native samples (Fig. 3). These findings were not surprising, as brevetoxins are primarily bound to circulating high, low, and very-low density lipoproteins in plasma (and also likely to components of the ILE), which were mostly removed from the sample after ultracentrifugation^52,53^. The exact mechanism of action of ILE-induced recovery is unknown, but is likely related to the “lipid sink” effect whereby toxins are pulled from target tissues with high concentrations and sequestered into the lipid component of the blood rendering them biologically inactive^22,23^. Other theories include increased ATP synthesis or calcium concentrations in cardiomyocytes following ILE delivery, but these mechanisms may be more likely with other drugs, including calcium channel blocking agents^21,23,26^. The ultra centrifugation results presented here suggest that, despite ILE therapy, a number of brevetoxins in the blood plasma are free/unbound, potentially allowing these brevetoxins to distribute to tissues for biological action at voltage-gated sodium channels in nervous, cardiac, or muscle tissue^52,53^. All but one sea turtle included in this study received a dose of 25 mg ILE/kg body mass. Based on these findings, we suggest that a higher dosage can be used (up to 50–100 mg/kg) to ensure more rapid detoxification of circulating free brevetoxins. This proposed higher dose was shown to be safe using slow administration (~ 1 mL/min) in one juvenile Kemp’s ridley from this study, red-eared sliders experimentally dosed with PbTx-3 and ILE^28^, and sea turtles rehabilitating at Loggerhead Marinelife Center (Juno Beach, FL, USA) experiencing toxicoses of unknown origin^54^. Removal of lipids here served as a means to better understand brevetoxin physiology, especially in regards to ILE therapy. A number of factors are known to interact with ELISA protocols including salinity, pH, and the presence of lipids in samples; however, the ELISA used here is not impacted by the lipids^55^.

Survival rate of all sea turtles in this study that received ILE was 94% (17/18), a significant improvement over the mean 47% (46/99) survival rate documented in other studies that used SSC and/or dehydration therapy to treat turtles experiencing brevetoxicosis (Table 3)^5,19,32^. The successful use of ILE to treat neurotoxicity has been documented in numerous other species^56^ including rats treated with methamphetamine^57^, domestic dogs with bromethalin toxicosis^44^, domestic cats with permethrin toxicosis^58^, African lions (*Panthera leo*) with ivermectin toxicosis^24^, green turtles exposed to domoic acid^30^, and double-crested cormorants (*Phalacrocorax auritus*) experiencing brevetoxicosis^59^. ﻿Only one sea turtle patient – a juvenile Kemp’s ridley of adequate body condition – did not survive after receiving ILE treatment. Upon intake, this animal presented with a quiet mentation, moderate dehydration, hypercalcemia, hyperkalemia, delayed blink reflex, bulging eyes, an inability to hold up its head and move its flippers, and an absent clasper reflex. In addition to ILE, the patient also received an intravenous dextrose bolus followed by sodium bicarbonate in an attempt to correct the severe hyperkalemia. Despite treatment, the animal succumbed to brevetoxicosis after < 12 h in rehabilitation. Interestingly, this animal had the third highest ELISA plasma brevetoxin concentration on intake; however, the relationship between brevetoxin exposure intensity and duration and tissue concentrations is unclear^12^, as previous studies have shown no correlation between plasma brevetoxin concentrations and survival^5^. Thresholds for the onset of symptoms associated with brevetoxicosis likely do not exist as previously suggested^16^, as we documented a wide range of concentrations on intake (1.6–93.4 ng PbTx-3/mL). In loggerheads receiving SSC in a separate study, those that survived eliminated nearly 80% of toxins by day 20, whereas those that died had eliminated just 40–60% in the same time frame^5^. Therefore, the severity of neurological symptoms and the presence of secondary conditions/diseases (e.g. metabolic acidosis, spirorchiid infection, pneumonia, malnutrition), in addition to timing of exposure and potential re-metabolism of brevetoxin compounds into circulation, likely all impact survivorship^5,12^.

Other treatments for brevetoxicosis have shown mixed results. Cholestyramine, a resin that binds with bile acids and prevents bile reabsorption^60^, showed no effect on clinical symptoms of brevetoxicosis in red-eared sliders at the administered doses (20 mg/kg and 50 mg/kg); however, non-significant declines in brevetoxin concentrations in brain, heart, fat, feces, kidney, and liver were observed in comparison to control turtles receiving no cholestyramine. The authors attributed the lack of findings to low sample sizes and low or too few dosages^28^. Another drug, the diuretic furosemide, was used successfully to treat brevetoxicosis in loggerheads, in addition to withholding fluids for the first 2–3 days of rehabilitation and administration of other treatments for secondary conditions (e.g. diphenhydramine to reduce conjunctival edema)^19^. Using furosemide, an increase in survival was achieved compared to SSC, whereby 71% (5/7) of loggerheads survived to release using dehydration therapy (e.g. furosemide) compared to 12% (2/17) using a combination of SSC, mannitol, and activated charcoal^19^. Neurological symptoms improved with the use of furosemide over the course of 1–3 days, with turtles eating on their own within 3–5 days^19^. Despite the potential success with other treatment methods, ILE therapy appears to lead to the fastest reduction in neurological symptoms in addition to an increase in survival rate^19^. As safety for ILE had not been previously established in sea turtles, a lower dose was selected (25 mg/kg) for treatment. However, based on the study in red-eared sliders^28^, and on more recent clinical experience in sea turtles^31^, we recommend providing a higher dose (up to 50–100 mg/kg) to sea turtles stranding with symptoms of brevetoxicosis.

Time from admission to release ranged from 14–62 days in turtles included in this study, which is similar or slightly lower than typical in comparison to most other studies; however, release is often delayed, despite full patient recovery, due to the presence of active blooms in areas where turtles are rehabilitated^5,19,32^. For example, loggerheads stranding during the red tide blooms of 2005–2006 provided with SSC and dehydration therapy were ready for release ~ 30–45 days post-admission, but due to the presence of a large-scale, active red tide bloom at time(s) of recovery, release times for all species ranged from 41–198 days^19^. Release times of 16 sea turtles from CROW that received SSC was 20 ± 6 days on average, similar to our study. Therefore, while symptoms are eliminated faster in turtles receiving ILE, full patient recovery and release are dependent on additional factors such as the development of secondary conditions, environmental factors including bloom severity and the presence of active blooms, and differences in veterinary care/release protocols^19^. It has also been suggested that loggerheads with brevetoxicosis are more difficult to treat in comparison to Kemp’s ridleys and green turtles^19^. Any even slight reductions in time to recovery and release will allow for the saving of resources, which is especially important for wildlife rehabilitation facilities whose resources are already stretched thin^61^.

When red tide blooms become extensive, increases in sea turtle (and other marine animal) strandings occur. Using previous techniques (e.g. SSC), turtles often succumbed to brevetoxicosis due to lack of effective and fast-acting therapies. Numerous turtles that would otherwise perish or require extensive periods in rehabilitation due to brevetoxicosis can now be successfully rehabilitated and released both with greater success and at increased rates. These types of fast-acting treatment plans are necessary for rehabilitation facilities that are already resource-limited. Intravenous lipid emulsion could also be tested in organisms exposed to other neuro- and/or lipophilic toxins, as symptom reduction has been shown in green turtles exposed to domoic acid and given ILE (although domoic acid is hydrophilic^62^)^30^. The use of ILE showed little to no risks with the sea turtles treated in this study; therefore, this therapy has the potential to increase survival and reduce rehabilitation time of sea turtles, and numerous other marine animals (e.g. seabirds) entering rehabilitation facilities worldwide.

## Methods

### Red tides: 2017–2019

*Karenia brevis* cells were first detected in October 2017 at very low to low concentrations on Florida’s west coast in Sarasota and Manatee Counties, shortly after Hurricane Irma made landfall in Florida. A red tide bloom (> 100,000 K*. brevis* cells/L) then began in November 2017 and persisted until February 2019. This bloom was one of the worst on record and reached its peak from September–November 2018, where 12–20 counties on Florida’s east and west coasts experienced red tide conditions, with a state of emergency declared in August 2018. Over 500 sea turtles stranded as a result of the red tide^39^. Again, in August 2019, very low to low concentrations of *K. brevis* were detected in offshore waters of Sarasota and Manatee Counties, with the bloom forming and persisting from September–December 2019^37^.

### Sample collection and animal care

Loggerhead, Kemp’s ridley, and green sea turtles with suspected brevetoxicosis were included in this study. Upon stranding on Florida’s west coast, these animals were admitted to CROW (26.443789°N, -82.115413°W) in Sanibel Island, FL, USA for veterinary care (i.e. SSC including removing turtles from the source of brevetoxins, placing them in toxin-free water, providing them with subcutaneous fluids, and gavage feeding). For each turtle, complete physical and neurological exams were performed and included evaluation of life-stage class, sex, mass and SCL on intake, body condition score, blink reflex, presence/absence of conjunctival swelling, ability to hold up head and flippers, circling behavior, clasper reflex, vent tone, dehydration, and demeanor. Neurologic exams were repeated daily until symptoms resolved. A small blood sample (up to 2 ml) was collected aseptically upon admission to rehabilitation (time point = 0 h). Following venipuncture, 25 mg ILE/kg body mass (Intralipid® 20%, Baxter Healthcare Corp., Deerfield, IL, USA) was administered (one Kemp’s ridley received a dosage of 100 mg ILE/kg body mass) using infusion pumps. Subsequent blood samples were collected from each turtle 1 h, 2 h, 6 h, 24 h, 48 h, 72 h, and 168 h post-ILE administration and then weekly after that as necessary (for up to nine weeks). Blood samples were subsequently placed into lithium heparin vacutainers and centrifuged for 10 min at 1534 g (3500 rpm) using a LW Scientific E8 Combination Centrifuge (LW Scientific, Lawrenceville, GA, USA). The resulting plasma was separated and stored frozen in an ultralow freezer until analysis for 75–633 d (mean ± SD: 457 ± 174 d) after collection. Samples with moderate or marked hemolysis (2–3 +)^63^ were removed from sample analyses.

In an effort to understand the potential impacts of lipemia on the ELISA, five lipemic samples were chosen, separated into two aliquots: a native sample and a sample that underwent ultracentrifugation (15,596 g/15,000 rpm) using an Oxford BenchMate C12V High Speed Microcentrifuge. This method has been shown to eliminate lipids from serum and plasma samples^64^. Brevetoxin concentrations from both samples were then determined using the ELISA procedure described below.

### Brevetoxin analysis: ELISA

Plasma brevetoxin concentrations were analyzed using a competitive ELISA from MARBIONC (Wilmington, NC, USA)^55^. This assay detects and measures brevetoxin congeners with the dominant (80%) B-type backbone (PbTx-2, 3, 5, 6, 8, 9). Congeners with the A-type backbone (PbTx-1, 7, 10) are also recognized, but at reduced affinities^5^; therefore, the resulting concentrations from the ELISA are a sum of the detected brevetoxin congeners reported as ng PbTx-3/mL. The assay procedure followed manufacturer’s instructions using the provided control, antigen, and antibodies^13^. Brevetoxin concentrations were determined using absorbance readings (at 450 nm) using a BioTek® H1M Synergy™ microplate reader (Winooski, VT, USA). Concentrations were calculated using a standard curve of the brevetoxin control.

### Statistical analyses

Statistical analyses were conducted using MedCalc (version 19.5.3, Ostend, Belgium) and R^65^. Measures of central tendency and range are reported for brevetoxin concentrations in plasma upon admission and for each subsequent time point (0 h, 1 h, 2 h, 6 h, 24 h, 48 h, 72 h, 168 h, etc.) for each species. A Kruskal–Wallis test was used to determine species differences between brevetoxin concentrations upon admission to rehab.

For statistical purposes, we combined sex and maturity status as one factor with three levels: immature, mature male, and mature female. We tested the effect and interactions of species, sex/maturity, and time point on brevetoxin concentrations using linear mixed-effect (LME) models. Because individuals may respond differently to ILE, we included each individual specimen as a random factor in two models, one with each individual having their own intercept (1|individual) and the other with each individual having their own slope (1 + time point|individual). We used ANOVA to compare the fits of these two models and Akaike’s Information Criteria (AIC) for parsimonious model selection. To generate *P* values for interactions and main effects, we used ANOVA with type III sum of squares. For pairwise comparisons between each time point and brevetoxin concentrations, we adjusted *P* values for multiple comparisons using Tukey’s method. These mixed-effect model analyses were first done with all three species included in the model, and then for each species separately, thus excluding “species” as a main effect in the latter models. The LME model was fitted using the packages ‘lme4,’^66^, ‘car,’^67^, and ‘emmeans’^68^.

Clearance rates of plasma brevetoxins determined by the ELISA in loggerheads and Kemp’s ridleys receiving ILE were compared to clearance rates in turtles that were provided with SSC^5^. Data^5^ were estimated using the online software WebPlotDigitizer verson 4.3^69^. Because the methods and timing of collection differed between the two studies, the results were not compared statistically. Instead, we established a line-of-best fit for the decline in brevetoxin concentrations for both studies and visualized the trends.

Brevetoxin concentrations in five lipemic native (i.e. processed as-is) samples were compared to those where the lipids were removed by ultracentrifugation using a paired samples t-test. Fisher’s exact tests were used to compare survival rate of turtles undergoing ILE therapy to those that were previously treated using SSC or furosemide across sea turtle rehabilitation facilities^5,19,32^. Lastly, a Welch’s t-test was carried out (as the variance between the two groups was unequal) to determine any statistically significant differences in rehabilitation time between turtles treated with ILE and those provided with SSC.

### Ethics approval and consent to participate

Our work was authorized under Florida Fish and Wildlife Conservation Commission Marine Turtle Permits #146 and #211 and Loggerhead Marinelife Center’s Animal Welfare Committee Protocol #2019–001; we performed all animal handling and collection in accordance with these relevant guidelines and regulations. This study also complies with the ARRIVE guidelines.

## Supplementary Information

Supplementary Information.
