# Supplementary material for: Use of intravenous lipid emulsion therapy as a novel treatment for brevetoxicosis in sea turtles
Source: Sci Rep. 2021 Dec 17;11:24162. doi: 10.1038/s41598-021-03550-y (PMC8683444; doi:10.1038/s41598-021-03550-y)
Supplement: Supplementary file 1 — Supplementary Information. [file 41598_2021_3550_MOESM1_ESM.docx]

| **Supplemental Table 1.** Species, stranding location, rehabilitation time, life-stage class, gender, mass on intake, body condition score (BCS), clinical symptoms of brevetoxicosis, initial brevetoxin concentrations, and additional treatments in stranded sea turtles experiencing brevetoxicosis that received intravenous lipid emulsion (ILE). | | | | | | | | | |
| --- | --- | --- | --- | --- | --- | --- | --- | --- | --- |
| Species | Stranding location | Dates in rehab | Life-stage | Gender | Intake mass (kg) | BCS | Clinical symptoms upon admission | Initial PbTx [ng/mL] | Additional Tx |
| *L. kempii*  18253  18-744 | Sanibel | 27 Mar–10 Apr 2018 | Adult | F | 32.3 | Moderate | Blink reflex: delayed  Bulging eyes: absent  Able to hold up head: weak  Able to move flippers: weak  Circling: absent  Clasper reflex: present  Vent tone: present  Dehydration: moderate  Mentation: depressed | 7.7 | Iron/B12 |
| *C. caretta*  18316  18-1446 | NA | 28 Apr–15 May 2018 | Sub-adult | U | 59.0 | Moderate | Blink reflex: present  Bulging eyes: present  Able to hold up head: present  Able to move flippers: present  Circling: unknown  Clasper reflex: present  Vent tone: present  Dehydration: mild  Mentation: quiet | 23.9 | Iron/B12  Praziquantel |
| *L. kempii*  18320  18-1463 | Goodland | 29 Apr–25 May 2018 | Juvenile | U | 14.4 | Thin | Blink reflex: present  Bulging eyes: absent  Able to hold up head: present  Able to move flippers: present  Circling: absent  Clasper reflex: present  Vent tone: present  Dehydration: none  Mentation: alert | 37.1 | NA |
| *C. caretta*  18370  18-2069 | Fort Myers | 29 May–12 Jun 2018 | Juvenile | U | 60.5 | Moderate | Blink reflex: present  Bulging eyes: present  Able to hold up head: present  Able to move flippers: present  Circling: absent  Clasper reflex: absent  Vent tone: present  Dehydration: NA  Mentation: quiet | 50.7 | Ceftazidime |
| *C. caretta*  18384  18-2170 | Sanibel | 4 Jun–28 Jun 2018 | Adult | M | 108.3 | Moderate | Blink reflex: present  Bulging eyes: absent  Able to hold up head: present  Able to move flippers: present  Circling: absent  Clasper reflex: present  Vent tone: present  Dehydration: none  Mentation: alert | 50.1 | Ceftazidime  Prazinquantel |

| **Supplemental Table 1 continued.** | | | | | | | | | |
| --- | --- | --- | --- | --- | --- | --- | --- | --- | --- |
| *L. kempii*  18422  18-2539 | Boca Grande | 28 Jun–16 Jul 2018 | Juvenile | U | 8.0 | Moderate | Blink reflex: present  Bulging eyes: absent  Able to hold up head: weakly  Able to move flippers: weakly  Circling: unknown  Clasper reflex: weak to absent  Vent tone: present  Dehydration: moderate  Mentation: quiet | 93.4 | Ceftazidime  Iron/B12  Saline |
| *C. caretta*  18467  18-2595 | Naples | 1 Jul–27 Jul 2018 | Sub-adult | U | 47.8 | Thin | Blink reflex: present  Bulging eyes: absent  Able to hold up head: weak  Able to move flippers: weak  Circling: unknown  Clasper reflex: present  Vent tone: present  Dehydration: moderate  Mentation: quiet | 1.6 | Ceftazidime  Iron/B12  Saline |
| *C. caretta*  18475  18-2895 | Sanibel | 20 Jul–13 Aug 2018 | Adult | U | 69.5 | Moderate | Blink reflex: present  Bulging eyes: present  Able to hold up head: present  Able to move flippers: present  Circling: absent  Clasper reflex: present  Vent tone: present  Dehydration: mild  Mentation: quiet | 36.5 | Ceftazidime  Saline |
| *C. caretta*  18481  18-2983 | Captiva | 26 Jul–22 Aug 2018 | Adult | M | 108.5 | Thin | Blink reflex: present  Bulging eyes: absent  Able to hold up head: present  Able to move flippers: present  Circling: absent  Clasper reflex: present  Vent tone: present  Dehydration: mild  Mentation: alert | 12.6 | Ceftazidime |
| *C. caretta*  18483  18-2988 | Captiva | 26 Jul–26 Sep 2018 | Adult | M | 111.6 | Thin | Blink reflex: present  Bulging eyes: present  Able to hold up head: present  Able to move flippers: present  Circling: absent  Clasper reflex: present  Vent tone: present  Dehydration: mild  Mentation: alert | 19.9 | Ceftazidime  Iron/B12  Saline |

| **Supplemental Table 1 continued.** | | | | | | | | | |
| --- | --- | --- | --- | --- | --- | --- | --- | --- | --- |
| *C. caretta*  18531  18-3223 | Naples | 14 Aug–26 Sep 2018 | Adult | M | 94.0 | Moderate | Blink reflex: present  Bulging eyes: present  Able to hold up head: present  Able to move flippers: present  Circling: absent  Clasper reflex: NA  Vent tone: absent  Dehydration: mild  Mentation: alert | 35.5 | Ceftazidime  Iron/B12  Saline |
| *C. caretta*  18532  18-3241 | Sanibel | 15 Aug–3 Oct 2018 | Adult | F | 132.4 | Good | Blink reflex: present  Bulging eyes: present  Able to hold up head: present  Able to move flippers: present  Circling: absent  Clasper reflex: present  Vent tone: present  Dehydration: mild  Mentation: alert | 64.6 | Ceftazidime  Crystalloid fluid therapy  Saline |
| *C. mydas*  18534  18-3277 | Sanibel | 18 Aug–6 Sep 2018 | Juvenile | U | 20.2 | Moderate | Blink reflex: present  Bulging eyes: absent  Able to hold up head: present  Able to move flippers: present  Circling: absent  Clasper reflex: present  Vent tone: present  Dehydration: mild  Mentation: alert | 13.1 | Ceftazidime  Saline |
| *C. mydas*  18540  18-3314 | Fort Myers | 21 Aug–6 Sep 2018 | Juvenile | U | 32.9 | Good | Blink reflex: present  Bulging eyes: absent  Able to hold up head: present  Able to move flippers: present  Circling: absent  Clasper reflex: present  Vent tone: present  Dehydration: none  Mentation: quiet | 4.9 | Ceftazidime  Saline |
| *C. mydas*  19500  19-4213 | Marco Island | 9 Oct–28 Oct 2019 | Juvenile | U | 10.9 | Thin | Blink reflex: present  Bulging eyes: absent  Able to hold up head: weak  Able to move flippers: weak  Circling: floating  Clasper reflex: present  Vent tone: present  Dehydration: moderate  Mentation: quiet | 6.8 | Ceftazidime  Saline |

| **Supplemental Table 1 continued.** | | | | | | | | | |
| --- | --- | --- | --- | --- | --- | --- | --- | --- | --- |
| *L. kempii*  19514  19-4280 | Marco Island | 14 Oct–15 Oct^*^ 2019  ^*^deceased | Juvenile | U | 16.6 | Moderate | Blink reflex: delayed  Bulging eyes: present  Able to hold up head: weak  Able to move flippers: weak  Circling: NA  Clasper reflex: absent  Vent tone: present  Dehydration: moderate  Mentation: quiet | 62.6 | Dextrose  Sodium bicarbonate |
| *C. mydas*  19613  19-4724 | Pine Island | 12 Nov–26 Nov 2019 | Juvenile | U | 6.2 | Moderate | Blink reflex: present  Bulging eyes: present  Able to hold up head: present  Able to move flippers: present  Circling: present  Clasper reflex: present  Vent tone: present  Dehydration: mild  Mentation: quiet | 15.3 | Saline |
| *L. kempii*  19633  19-4900 | Marco Island | 27 Nov–23 Dec 2019 | Juvenile | U | 7.9 | Thin | Blink reflex: present  Bulging eyes: present  Able to hold up head: weak  Able to move flippers: present  Circling: unknown  Clasper reflex: present  Vent tone: absent  Dehydration: moderate  Mentation: quiet | 5.0 | Ceftazidime  Iron/B12  Saline |

| **Supplemental Table 2.** Time-point comparisons for linear mixed-effect model with all three sea turtle species (loggerhead, *Caretta caretta*; Kemp’s ridley, *Lepidochelys kempii*; green turtle, *Chelonia mydas*) included. These results are from the most parsimonious model that included a random effect of individuals with their own slope (1 + time point\|individual). Significant comparisons (*P* < 0.05) are bolded. All *P* values are adjusted for multiple comparisons using Tukey method. | | | | | |
| --- | --- | --- | --- | --- | --- |
| Contrast | Estimate | Standard error | df | t | *P* |
| 1*2 | 8.069 | 2.65 | 107.8 | 3.041 | 0.083 |
| 1*3 | 7.758 | 2.65 | 107.9 | 2.923 | 0.112 |
| 1*4 | 6.994 | 2.71 | 108.3 | 2.585 | 0.238 |
| 1*5 | 12.921 | 2.72 | 110.7 | 4.748 | **<0.001** |
| 1*6 | 15.592 | 2.78 | 118.2 | 5.599 | **<0.001** |
| 1*7 | 17.397 | 2.93 | 123.2 | 5.933 | **<0.001** |
| 1*8 | 23.179 | 3.47 | 105.7 | 6.682 | **<0.001** |
| 1*9 | 23.81 | 4.72 | 46.5 | 5.041 | **<0.001** |
| 1*10 | 25.993 | 6.53 | 26.4 | 3.98 | **0.015** |
| 2*3 | -0.311 | 2.65 | 107.8 | -0.117 | 1.000 |
| 2*4 | -1.075 | 2.71 | 108.3 | -0.397 | 1.000 |
| 2*5 | 4.852 | 2.72 | 110.5 | 1.784 | 0.744 |
| 2*6 | 7.522 | 2.78 | 118.1 | 2.703 | 0.185 |
| 2*7 | 9.328 | 2.93 | 123 | 3.184 | 0.056 |
| 2*8 | 15.109 | 3.46 | 106.2 | 4.362 | **0.001** |
| 2*9 | 15.741 | 4.72 | 46.6 | 3.337 | **0.048** |
| 2*10 | 17.924 | 6.53 | 26.4 | 2.746 | 0.206 |
| 3*4 | -0.764 | 2.7 | 108.1 | -0.282 | 1.000 |
| 3*5 | 5.163 | 2.72 | 109.9 | 1.901 | 0.668 |
| 3*6 | 7.834 | 2.77 | 117.1 | 2.825 | 0.140 |
| 3*7 | 9.639 | 2.92 | 122.8 | 3.299 | **0.040** |
| 3*8 | 15.421 | 3.45 | 107.5 | 4.467 | **<0.001** |
| 3*9 | 16.052 | 4.7 | 46.8 | 3.413 | **0.039** |
| 3*10 | 18.235 | 6.51 | 26.4 | 2.801 | 0.187 |
| 4*5 | 5.927 | 2.74 | 108.9 | 2.165 | 0.487 |
| 4*6 | 8.598 | 2.79 | 115.4 | 3.082 | 0.074 |
| 4*7 | 10.403 | 2.94 | 121.4 | 3.544 | **0.019** |
| 4*8 | 16.185 | 3.45 | 111.1 | 4.685 | **<0.001** |
| 4*9 | 16.816 | 4.7 | 48.5 | 3.581 | **0.025** |
| 4*10 | 18.999 | 6.5 | 26.8 | 2.923 | 0.148 |
| 5*6 | 2.67 | 2.75 | 111.1 | 0.97 | 0.993 |
| 5*7 | 4.476 | 2.89 | 117.8 | 1.547 | 0.870 |
| 5*8 | 10.257 | 3.37 | 119.4 | 3.043 | 0.082 |
| 5*9 | 10.889 | 4.58 | 51.6 | 2.376 | 0.361 |
| 5*10 | 13.072 | 6.39 | 27.2 | 2.047 | 0.576 |
| 6*7 | 1.805 | 2.86 | 114.2 | 0.631 | 1.000 |
| 6*8 | 7.587 | 3.26 | 127.1 | 2.326 | 0.380 |
| 6*9 | 8.219 | 4.42 | 56.1 | 1.86 | 0.695 |
| 6*10 | 10.401 | 6.21 | 27.6 | 1.674 | 0.800 |
| 7*8 | 5.782 | 3.22 | 127 | 1.795 | 0.737 |
| 7*9 | 6.413 | 4.32 | 68.3 | 1.485 | 0.894 |
| 7*10 | 8.596 | 6.11 | 30.4 | 1.408 | 0.916 |
| 8*9 | 0.632 | 3.98 | 99.5 | 0.159 | 1.000 |
| 8*10 | 2.814 | 5.68 | 37.3 | 0.495 | 1.000 |
| 9*10 | 2.183 | 5.21 | 84.4 | 0.419 | 1.000 |

| **Supplemental Table 3.** Time-point comparisons for linear mixed-effect model for loggerhead sea turtles (*Caretta caretta*). These results are from the most parsimonious model that included a random effect of individuals with their own slope (1 + time point\|individual). Significant comparisons (*P* < 0.05) are bolded. All *P* values are adjusted for multiple comparisons using Tukey method. | | | | | |
| --- | --- | --- | --- | --- | --- |
| Contrast | Estimate | Standard error | df | t | *P* |
| 1*2 | 7.287 | 3.26 | 62 | 2.234 | 0.446 |
| 1*3 | 8.524 | 3.26 | 62 | 2.613 | 0.234 |
| 1*4 | 6.871 | 3.26 | 62 | 2.106 | 0.530 |
| 1*5 | 14.101 | 3.26 | 62 | 4.323 | **0.002** |
| 1*6 | 14.716 | 3.26 | 62 | 4.511 | **0.001** |
| 1*7 | 17.111 | 3.26 | 62 | 5.245 | **<0.001** |
| 1*8 | 24.389 | 3.52 | 62.1 | 6.933 | **<0.001** |
| 1*9 | 22.411 | 3.7 | 62.1 | 6.065 | **<0.001** |
| 1*10 | 25.899 | 4.25 | 62.2 | 6.091 | **<0.001** |
| 2*3 | 1.238 | 3.26 | 62 | 0.379 | 1.000 |
| 2*4 | -0.416 | 3.26 | 62 | -0.127 | 1.000 |
| 2*5 | 6.814 | 3.26 | 62 | 2.089 | 0.542 |
| 2*6 | 7.429 | 3.26 | 62 | 2.277 | 0.418 |
| 2*7 | 9.824 | 3.26 | 62 | 3.012 | 0.099 |
| 2*8 | 17.103 | 3.52 | 62.1 | 4.862 | **<0.001** |
| 2*9 | 15.125 | 3.7 | 62.1 | 4.093 | **0.005** |
| 2*10 | 18.612 | 4.25 | 62.2 | 4.377 | **0.002** |
| 3*4 | -1.653 | 3.26 | 62 | -0.507 | 1.000 |
| 3*5 | 5.577 | 3.26 | 62 | 1.709 | 0.786 |
| 3*6 | 6.191 | 3.26 | 62 | 1.898 | 0.670 |
| 3*7 | 8.587 | 3.26 | 62 | 2.632 | 0.225 |
| 3*8 | 15.865 | 3.52 | 62.1 | 4.51 | **0.001** |
| 3*9 | 13.887 | 3.7 | 62.1 | 3.758 | **0.013** |
| 3*10 | 17.375 | 4.25 | 62.2 | 4.086 | **0.005** |
| 4*5 | 7.23 | 3.26 | 62 | 2.216 | 0.457 |
| 4*6 | 7.844 | 3.26 | 62 | 2.405 | 0.341 |
| 4*7 | 10.24 | 3.26 | 62 | 3.139 | 0.072 |
| 4*8 | 17.518 | 3.52 | 62.1 | 4.98 | **<0.001** |
| 4*9 | 15.54 | 3.7 | 62.1 | 4.205 | **0.003** |
| 4*10 | 19.028 | 4.25 | 62.2 | 4.475 | **0.001** |
| 5*6 | 0.614 | 3.26 | 62 | 0.188 | 1.000 |
| 5*7 | 3.01 | 3.26 | 62 | 0.923 | 0.995 |
| 5*8 | 10.288 | 3.52 | 62.1 | 2.925 | 0.121 |
| 5*9 | 8.31 | 3.7 | 62.1 | 2.249 | 0.436 |
| 5*10 | 11.798 | 4.25 | 62.2 | 2.774 | 0.168 |
| 6*7 | 2.396 | 3.26 | 62 | 0.734 | 0.999 |
| 6*8 | 9.674 | 3.52 | 62.1 | 2.75 | 0.177 |
| 6*9 | 7.696 | 3.7 | 62.1 | 2.083 | 0.546 |
| 6*10 | 11.184 | 4.25 | 62.2 | 2.63 | 0.226 |
| 7*8 | 7.278 | 3.52 | 62.1 | 2.069 | 0.556 |
| 7*9 | 5.3 | 3.7 | 62.1 | 1.434 | 0.911 |
| 7*10 | 8.788 | 4.25 | 62.2 | 2.067 | 0.557 |
| 8*9 | -1.978 | 3.87 | 62 | -0.511 | 1.000 |
| 8*10 | 1.51 | 4.4 | 62.1 | 0.343 | 1.000 |
| 9*10 | 3.488 | 4.52 | 62.1 | 0.772 | 0.999 |

| **Supplemental Table 4.** Time-point comparisons for linear mixed-effect model for Kemp’s ridley sea turtles (*Lepidochelys kempii*). These results are from the most parsimonious model that included a random effect of individuals with their own slope (1 + time point\|individual). Significant comparisons (*P* < 0.05) are bolded. All *P* values are adjusted for multiple comparisons using Tukey method. | | | | | |
| --- | --- | --- | --- | --- | --- |
| Contrast | Estimate | Standard error | df | t | *P* |
| 1*2 | 9.95 | 10.5 | 23 | 0.95 | 0.993 |
| 1*3 | 7.29 | 10.5 | 23 | 0.696 | 0.999 |
| 1*4 | 4.82 | 11.3 | 23.2 | 0.426 | 1.000 |
| 1*5 | 13.05 | 11.3 | 23.2 | 1.154 | 0.973 |
| 1*6 | 20.94 | 11.3 | 23.2 | 1.852 | 0.698 |
| 1*7 | 27.56 | 12.4 | 23.3 | 2.217 | 0.473 |
| 1*8 | 38.62 | 12.4 | 23.3 | 3.108 | 0.109 |
| 1*9 | 40.89 | 12.4 | 23.3 | 3.29 | 0.075 |
| 1*10 | 23.06 | 18.9 | 23.3 | 1.219 | 0.962 |
| 2*3 | -2.66 | 10.5 | 23 | -0.254 | 1.000 |
| 2*4 | -5.13 | 11.3 | 23.2 | -0.454 | 1.000 |
| 2*5 | 3.1 | 11.3 | 23.2 | 0.274 | 1.000 |
| 2*6 | 10.99 | 11.3 | 23.2 | 0.972 | 0.991 |
| 2*7 | 17.61 | 12.4 | 23.3 | 1.417 | 0.910 |
| 2*8 | 28.68 | 12.4 | 23.3 | 2.307 | 0.420 |
| 2*9 | 30.94 | 12.4 | 23.3 | 2.49 | 0.323 |
| 2*10 | 13.11 | 18.9 | 23.3 | 0.693 | 0.999 |
| 3*4 | -2.47 | 11.3 | 23.2 | -0.219 | 1.000 |
| 3*5 | 5.76 | 11.3 | 23.2 | 0.509 | 1.000 |
| 3*6 | 13.65 | 11.3 | 23.2 | 1.207 | 0.964 |
| 3*7 | 20.27 | 12.4 | 23.3 | 1.631 | 0.820 |
| 3*8 | 31.33 | 12.4 | 23.3 | 2.521 | 0.308 |
| 3*9 | 33.6 | 12.4 | 23.3 | 2.704 | 0.229 |
| 3*10 | 15.77 | 18.9 | 23.3 | 0.834 | 0.997 |
| 4*5 | 8.23 | 11.7 | 23 | 0.703 | 0.999 |
| 4*6 | 16.12 | 11.7 | 23 | 1.378 | 0.922 |
| 4*7 | 22.74 | 12.8 | 23.1 | 1.778 | 0.742 |
| 4*8 | 33.8 | 12.8 | 23.1 | 2.643 | 0.254 |
| 4*9 | 36.07 | 12.8 | 23.1 | 2.82 | 0.187 |
| 4*10 | 18.24 | 19.1 | 23.2 | 0.952 | 0.992 |
| 5*6 | 7.89 | 11.7 | 23 | 0.674 | 0.999 |
| 5*7 | 14.51 | 12.8 | 23.1 | 1.135 | 0.975 |
| 5*8 | 25.58 | 12.8 | 23.1 | 2 | 0.607 |
| 5*9 | 27.84 | 12.8 | 23.1 | 2.177 | 0.498 |
| 5*10 | 10.01 | 19.1 | 23.2 | 0.523 | 1.000 |
| 6*7 | 6.62 | 12.8 | 23.1 | 0.518 | 1.000 |
| 6*8 | 17.69 | 12.8 | 23.1 | 1.383 | 0.921 |
| 6*9 | 19.95 | 12.8 | 23.1 | 1.56 | 0.854 |
| 6*10 | 2.12 | 19.1 | 23.2 | 0.111 | 1.000 |
| 7*8 | 11.06 | 13.5 | 23 | 0.819 | 0.998 |
| 7*9 | 13.33 | 13.5 | 23 | 0.987 | 0.990 |
| 7*10 | -4.5 | 19.6 | 23.2 | -0.229 | 1.000 |
| 8*9 | 2.27 | 13.5 | 23 | 0.168 | 1.000 |
| 8*10 | -15.57 | 19.6 | 23.2 | -0.793 | 0.998 |
| 9*10 | -17.83 | 19.6 | 23.2 | -0.908 | 0.995 |

| **Supplemental Table 5.** Time-point comparisons for linear mixed-effect model for green sea turtles (*Chelonia mydas*). These results are from the most parsimonious model that included a random effect of individuals with their own intercept (1\|individual). Significant comparisons (*P* < 0.05) are bolded. All *P* values are adjusted for multiple comparisons using Tukey method. | | | | | |
| --- | --- | --- | --- | --- | --- |
| Contrast | Estimate | Standard error | df | t | *P* |
| 1*2 | 7.4874 | 1.58 | 16.28 | 4.732 | **0.004** |
| 1*3 | 6.1876 | 1.59 | 16.4 | 3.896 | **0.021** |
| 1*4 | 8.3003 | 1.6 | 16.59 | 5.204 | **0.002** |
| 1*5 | 8.017 | 1.65 | 17.81 | 4.868 | **0.003** |
| 1*6 | 8.0276 | 1.82 | 18.51 | 4.401 | **0.006** |
| 1*7 | 7.509 | 1.83 | 19.52 | 4.106 | **0.011** |
| 1*8 | 9.2296 | 2.08 | 7.83 | 4.441 | **0.028** |
| 2*3 | -1.2998 | 1.59 | 16.39 | -0.819 | 0.989 |
| 2*4 | 0.8129 | 1.59 | 16.57 | 0.51 | 0.999 |
| 2*5 | 0.5296 | 1.65 | 17.78 | 0.322 | 1.000 |
| 2*6 | 0.5403 | 1.82 | 18.56 | 0.297 | 1.000 |
| 2*7 | 0.0216 | 1.83 | 19.51 | 0.012 | 1.000 |
| 2*8 | 1.7422 | 2.08 | 7.91 | 0.839 | 0.985 |
| 3*4 | 2.1127 | 1.58 | 16.34 | 1.333 | 0.873 |
| 3*5 | 1.8295 | 1.62 | 17.18 | 1.132 | 0.940 |
| 3*6 | 1.8401 | 1.76 | 19.03 | 1.044 | 0.961 |
| 3*7 | 1.3215 | 1.83 | 19.49 | 0.723 | 0.995 |
| 3*8 | 3.042 | 2.09 | 8.14 | 1.456 | 0.811 |
| 4*5 | -0.2832 | 1.6 | 16.83 | -0.177 | 1.000 |
| 4*6 | -0.2726 | 1.74 | 19.01 | -0.157 | 1.000 |
| 4*7 | -0.7912 | 1.82 | 19.33 | -0.435 | 1.000 |
| 4*8 | 0.9293 | 2.08 | 8.92 | 0.447 | 1.000 |
| 5*6 | 0.0106 | 1.65 | 17.81 | 0.006 | 1.000 |
| 5*7 | -0.508 | 1.84 | 18.99 | -0.276 | 1.000 |
| 5*8 | 1.2126 | 2.11 | 10.08 | 0.575 | 0.999 |
| 6*7 | -0.5186 | 1.96 | 18.9 | -0.264 | 1.000 |
| 6*8 | 1.2019 | 2.23 | 9.96 | 0.538 | 0.999 |
| 7*8 | 1.7206 | 1.92 | 19.07 | 0.894 | 0.983 |
